# Impacts of abundance and habitat area weighting in allocating species trends to habitats

**DOI:** 10.1007/s10661-025-14352-4

**Published:** 2025-07-09

**Authors:** Robin J. Pakeman

**Affiliations:** The James Hutton Institute, Craigiebuckler, Aberdeen, AB15 8QH UK

**Keywords:** Biodiversity trends, Bryophytes, Habitat trends, Lichens, Weighted geometric means

## Abstract

The dynamics of species depend on the management of their habitats. However, in the absence of good habitat monitoring data for many types of species, reliance has been placed on identifying habitats seeing marked changes in biodiversity through combining trends in their associated species into a habitat level metric. Several data sources on species occupancy, abundance within different habitats, and habitat area for two example taxa, bryophytes and lichens, were linked to assess how different methods of allocating existing species’ abundance trends to habitats influenced the habitat statistics. In general, trends through time were similar, but the method of allocation had an impact on the absolute values of the Distribution Index that summarises weighted occupancy. Allowing generalists to contribute equally to specialist species in a habitat gave higher values of habitat level Distribution Index than methods which weighted species according to abundance in that habitat and habitat area. There were also impacts on the analysis of long-term and short-term trend data, with the more complex methods, including abundance within habitats and extent of habitat, detecting more differences between habitats, and, for some habitats, changing positive trends for bryophytes to no significant trend or even negative for sparsely vegetated habitats. If species trend data is to be used for identifying habitats where biodiversity trends are marked, then it is clear that weighting species, such that their total weight across the analysis is the same, is necessary. Developing the precise means to achieve that needs careful thought and the creation of a robust method that works across different species groups, but using unweighted data could lead to erroneous conclusions as they are so dependent on the dynamics of widespread species.

## Introduction

The gold standard for assessing the ecological health of habitats is through repeat survey of different taxa (Buckland & Johnston, 2017; Teder et al., 2007). At the finest level of detail, this can show how individual species are performing in different habitats, but often there is an aggregation of species together to provide information at the habitat level. For instance, within the UK, this type of repeat survey data using specific methods such as the Breeding Bird Survey has shown population trends in farmland birds through time (Gregory et al., 2004, https://jncc.gov.uk/our-work/ukbi-c5-birds-of-the-wider-countryside-and-at-sea/) where farmland birds are identified using a pre-existing classification (Gibbons et al., 1993). Similarly, for butterflies, trends are calculated for different habitats from the Butterfly Monitoring Scheme (Brereton et al., 2011, https://jncc.gov.uk/our-work/ukbi-c6-insects-of-the-wider-countryside/), where butterflies are divided into species of the wider countryside and habitat specialists (Asher et al., 2001). Trends in the abundance of vascular plant species used to be the subject of similar repeat surveys, but at wider time intervals (Carey et al., 2008). Such repeat surveys are very powerful tools to identify habitats where management or other external drivers are having impacts on biodiversity and whether specialists (species with limited distribution across habitats, or narrow resource use, e.g. Devictor et al., 2008; Fischer & Stöcklin, 1997) or generalists (species with wide distributions across habitats and broad resource use) are behaving differently.

However, data for the majority of species is in the form of occupancy records, i.e. location data with limited metadata, usually taken as presence/absence data for an individual year of recording, contributed to databases such as the Global Biodiversity Information Facility (https://www.gbif.org/) or the UK-based National Biodiversity Network Atlas (https://nbnatlas.org/). Developing trends data from this type of information is possible but potentially computationally demanding (Boyd et al., 2023). However, unlike repeat survey data on abundance, this type of occupancy data is usually collected without associated habitat information, and it is therefore problematic to use this type of data to assess trends at the level of habitats. One attempt to address this used statistical models of habitat association based on probabilities of occurrence and land cover data to identify which species should contribute to which habitat trends. The method was developed for butterflies but is dependent on large numbers of records (c. 5000) to get an individual estimate of habitat association for each species (Redhead et al., 2016), but that means that only relatively common species contribute to a habitat-level estimate and these common species may not reflect the dynamics of rare species in different habitats (Zhu et al., 2025). This method was adopted in the UK’s State of Nature 2016 report which split species into those associated with a single habitat to look at trends for that habitat (Hayhow et al., 2016). However, species trends were allocated across multiple habitats, i.e. where there was a significant positive association between a species and a habitat (Burns et al., 2018). A similar approach but using existing habitat association data was used in a paper looking at trends in bryophyte and lichen occupancy in Scotland (Pakeman et al., 2022). The issue with this approach is that generalist species are used to calculate habitat trends across multiple habitats, whilst specialist species contribute only to a single habitat trend, so trend metrics become dominated by generalists and comparison between habitats is problematic (Burns et al. 2018, Pakeman et al., 2022). This could mean that conservation policies and actions may be based on information that has biases in it due to the dominance of generalist species in creating the habitat-level trends.

Both the statistical allocation of species to habitat (Redhead et al., 2016) and allocation using existing independent data on habitat association could be improved by making sure that the total weight for each species across all habitats are equal, so that generalists do not influence trends more than specialists. This could be done with expert judgement, but the analysis in this study focusses on three potential methods to equalise the weighting of species in the analysis and compares them with the unweighted analysis where a species contributes to a habitat trend if it is associated with that habitat.

The first method uses no further data but makes the simplest assumption that the weight of a species per habitat is a simple proportion, one divided by the number of habitats a species occurs in. So, a species occurring in three habitats has a weight of 0.333 in the analysis of trends for each habitat. This is simplistic but may be the only option where the ecology of a species is poorly known. The second option depends upon a measure of the relative abundance of species by habitat. In effect, asking a question that could be posed to experts, ‘if you visited habitat X, how frequently would you encounter species Y?’ So, a species would have its weight in the analysis split so that it had a higher weight for habitats where the species is more likely to be encountered. However, this option could be criticised in that whilst a species was commonly encountered in habitat X, if this habitat was relatively rare then that species’ trends might be more associated with habitats where it was less abundant than if that habitat was very common. A third method was used to investigate this; the weights based on abundance were adjusted by the relative area of each habitat. These three approaches address the range of potential complexity of factors that could make up an approach to weighting species. Further complexities could be added, for instance regarding the detectability of species in different habitats, but for the chosen taxa this was not seen as relevant.

The analysis was based on the same bryophyte and lichen trends used in Pakeman et al. (2022), but the weightings were adjusted using independent information from vegetation data and estimates of habitat area. This was used to ask the question ‘how does weighting species in the allocation of species’ trends to habitats change the results of simplistic analyses without weighting?’ The analyses were carried out for two datasets, (1) annual occupancy data for individual species and (2) their overall trends through time. This was done to highlight the potential differences between having more temporally detailed data available and only having trend data available.

## Methods

### Data sources

Four data sources were used in the analysis of annual occupancy data. (1) The annual occupancy scores for bryophytes and lichens used in constructing the Combined Biodiversity Indicator for Scotland (https://www.gov.scot/publications/development-combined-marine-terrestrial-biodiversity-indicator-scotland/, https://www.nature.scot/doc/marine-and-terrestrial-species-indicators-experimental-statistic) and in the analysis in Pakeman et al. (2022). They are created from ad hoc records from 10 km × 10 km grid squares for each species with enough records using Bayesian occupancy models (Barwell et al., 2021; Outhwaite et al., 2019). These data are used to calculate the trend data used in the analysis in the “Analysis of species trend data” Sect. (2) National Vegetation Classification (NVC, Rodwell, 1991a, 1991b, 1992, 1995, 2000) floristics tables which show which species occur in which NVC communities and include frequency and abundance measures (https://data.jncc.gov.uk/data/a407ebfc-2859-49cf-9710-1bde9c8e28c7/NVC-floristic-tables.xls). It should be noted that this limits the species in the analysis to those recorded during vegetation recording, i.e. ground living (terricolous) bryophytes and lichens. This limited the analysis to 163 bryophyte species and 58 lichen species compared to the 326 bryophyte and 437 lichen species in Pakeman et al. (2022). (3) A table of habitat correspondences which links NVC classes to EUNIS habitat types (European Nature Information System, https://hub.jncc.gov.uk/assets/9e70531b-5467-4136-88f6-3b3dd905b56d). (4) Area data of EUNIS habitats from the Habitat Map of Scotland (https://www.environment.gov.scot/our-environment/habitats-and-species/habitat-map-of-scotland/). There are issues with all the potential habitat maps, but the choice was limited to the Habitat Map of Scotland as it uses the EUNIS habitat types and because it delineates coastal and marine (saltmarsh) habitats separately rather than identifying them as grassland habitats.

### Data steps to calculate weightings

Within the floristic tables of the NVC, each species occurrence in the table is associated with a frequency (I representing a frequency of 1 to 20% of quadrats, II representing 21 to 40% etc., III, IV, V). Each species also has an abundance on the Domin Scale (a ten-point scale describing cover, Domin, 1923)—for the samples it occurred in. An approximate cover value per species per community was calculated as follows:

$$Cover estimate = Frequency midpoint \times Dominconverted cover proportion$$

where frequency was translated into midpoint values (e.g. class II → 30%) and Domin values converted using Currall (1987) where Percentage cover = (Domin score)^2.6^/4. Using the habitat correspondences in data source (3), a set of covers for each species per EUNIS Level 1 habitat type were calculated as the mean of the covers in the NVC communities contained within that habitat type. Species names were brought up to date using Taxonstand (Cayuela et al., 2021) in R ver. 4.1.2 (R Core Team, 2021).

Four types of weighting were created from these data:

- Unw—each occurrence of a species in all habitats was changed to a presence/absence measure (i.e. positive cover values replaced by one, such that weight for a specific habitat (*w*_Unw_) = 1). This was the approach used in Pakeman et al. (2022), but it means that generalists appear more in the analysis than specialists as they occur in more than one habitat.
- W-S—the weightings in Unw were divided by the number of habitat types that the species occurs in, i.e. they were standardised so that the total weight of each species summed to one across the habitats they occur in (*w*_W-S_ = 1/(no. habitats)). The weighting assumed generalists are distributed equally across the habitats they occur in.
- W-A—the weighting used the average cover per EUNIS level 1 habitat type and standardised them so that the total weight summed to one, hence all species have the same total weight. For a species that occurs in more than one habitat, it was split by its relative abundance across habitats so that the weighting reflects the relative abundance of species in a ‘sample’ of a habitat (*w*_W-A_ = abundance in h/sum of abundances across habitats).
- W-AH—the weights in W-A were adjusted by the total area of each EUNIS level 1 community – data source (4) so that w_W-AH_ = w_W-A_ * area of habitat/total area of habitats). This accounts for the possibility that the majority of a species’ population could be in a habitat that it occurs in at low frequency/abundance if that was a very common habitat, so trends in that species’ occupancy are likely to be driven by changes in the habitats where most of its population is found.

### Production of habitat trends

**Fig. 1:**
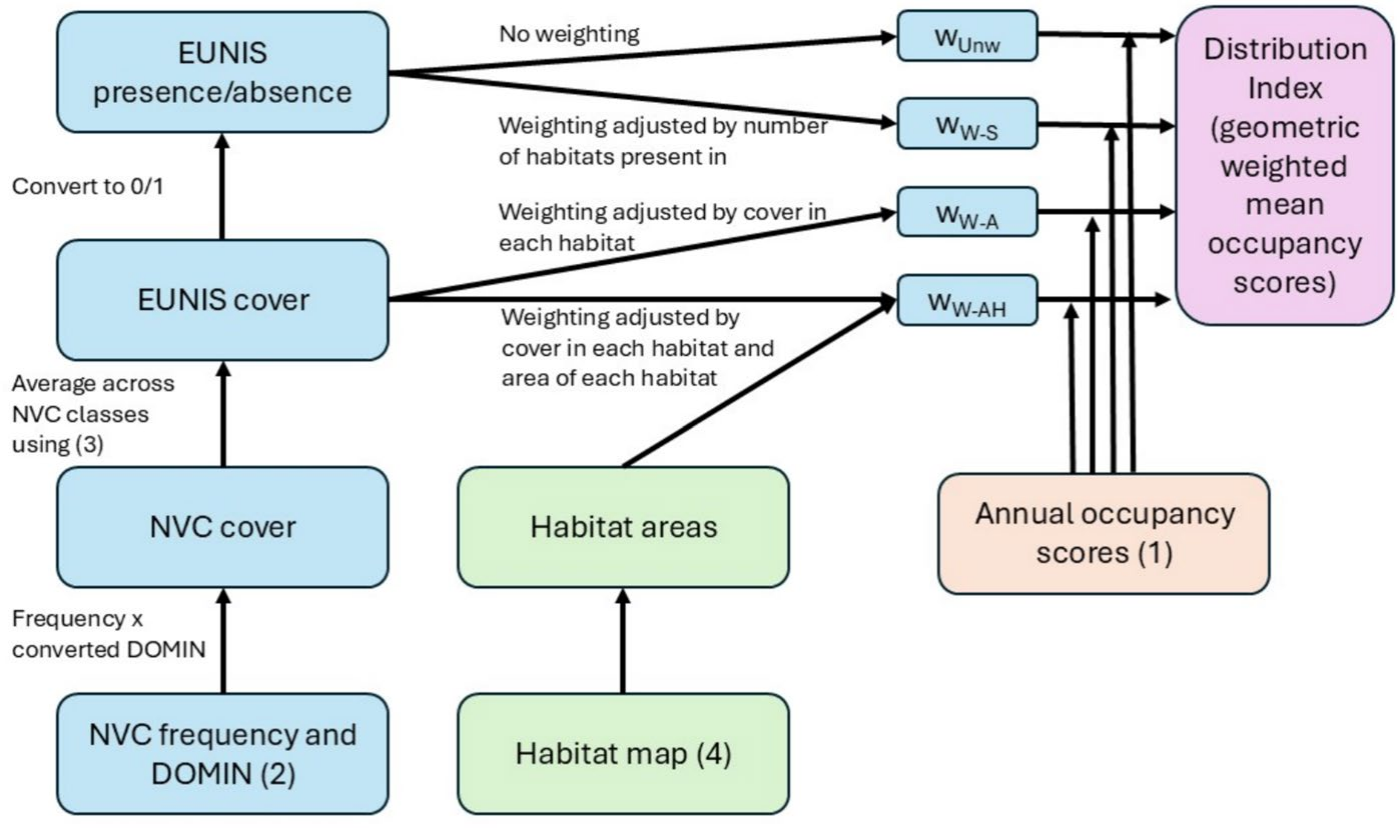
Workflow for creating the Distribution Index from habitat, land cover, and occupancy data. Numbers refer to the data sources listed in the “Data sources” section

In order to understand detailed temporal changes within habitats, trends in Distribution Index (weighted mean occupancy) across years for each habitat were produced by calculating the weighted geometric mean and standard deviations across annual occupancy scores of the species associated with that habitat type for each of the four weightings using the wgmean and wgsd functions from functClust in R (Jaillard et al., 2020). Mean Distribution Index scores for habitats were compared across weighting methods using a linear mixed model with year nested within habitat as the random effects using lme4 (Bates et al., 2015) and lmerTest (Kuznetsova et al., 2017). The workflow for producing the Distribution index is shown in Fig. 1.

### Analysis of species trend data

In some circumstances, only overall trend data may be available, so the analysis was repeated using trend data calculated from the same data. Long-term and short-term trends are produced as part of the State of Nature reports (Hayhow et al., 2019). These were analysed to assess how weighting affected the calculation of habitat level trends. Long-term trends were calculated for each species from the initial date of data availability (average 1972 for bryophytes and 1971 for lichens) to the penultimate year of data availability (2015). Short-term trends were calculated from 2005 to 2015. Trends are produced by creating a smoothed version of each species’ time-series using a thin plate spline model. Rates of change were calculated from the value in the penultimate year of a smoothed species’ time-series and that from the first year of the assessment period (Hayhow et al., 2019. Habitat specific trends were compared using a linear model of weighted mean trend ~ habitat × weighting method, with weights from the four methods of habitat allocation. Means were calculated using the emmeans package in R (Lenth, 2022).

## Results

### Habitat specific trends—bryophytes

In all habitats, the mean Distribution Index score was significantly and substantially higher for the unweighted method of occupancy estimation than for the three other methods (Table 1). Distribution Index estimates for the weighting method including both relative abundance and habitat area (W-AH) were substantially lower than for the other two weighting methods.

**Table 1:** Estimated differences and statistics from the mixed model between the unweighted Distribution Index scores (Intercept) and the three weighting methods W-S—weighting split evenly across habitats the species occurs in, W-A—weighting according to relative abundance of species per habitat, and W-AH—weighting according to relative abundance per habitat and the relative areas of habitat for bryophytes and lichens

	Estimate	Std. error	df	*t* value	Pr(>|*t*|)
Bryophytes					
Intercept (Unw)	0.286	0.019	9.084	15.39	< 0.001
W-S	− 0.042	0.002	1377	20.55	< 0.001
W-A	− 0.046	0.002	1377	22.16	< 0.001
W-AH	− 0.062	0.002	1377	30.25	< 0.001
Lichens					
Intercept (Unw)	0.234	0.040	5.011	5.902	0.002
W-S	− 0.010	0.002	807	4.772	< 0.001
W-A	− 0.024	0.002	807	11.61	< 0.001
W-AH	− 0.038	0.002	807	18.24	< 0.001

**Fig. 2:**
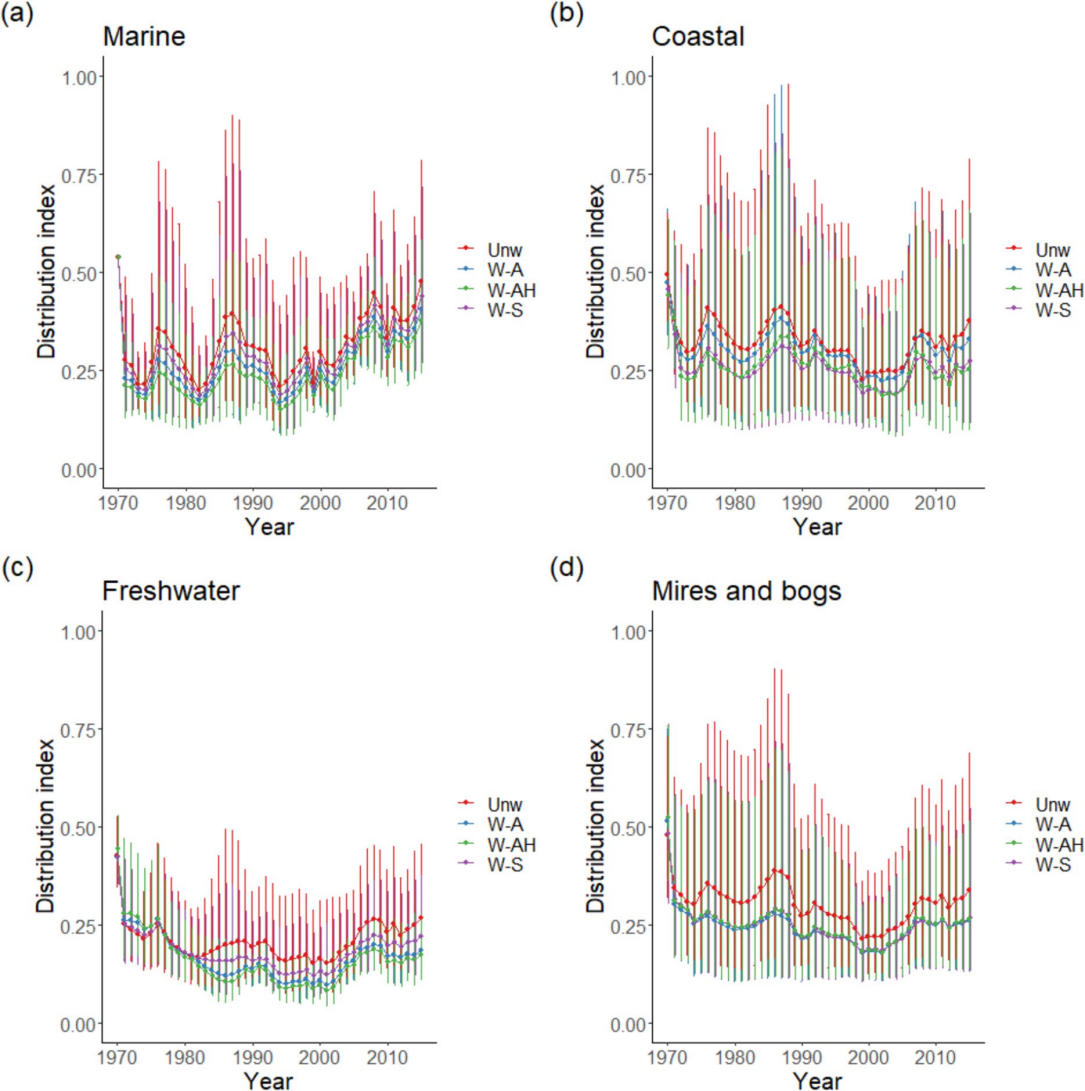

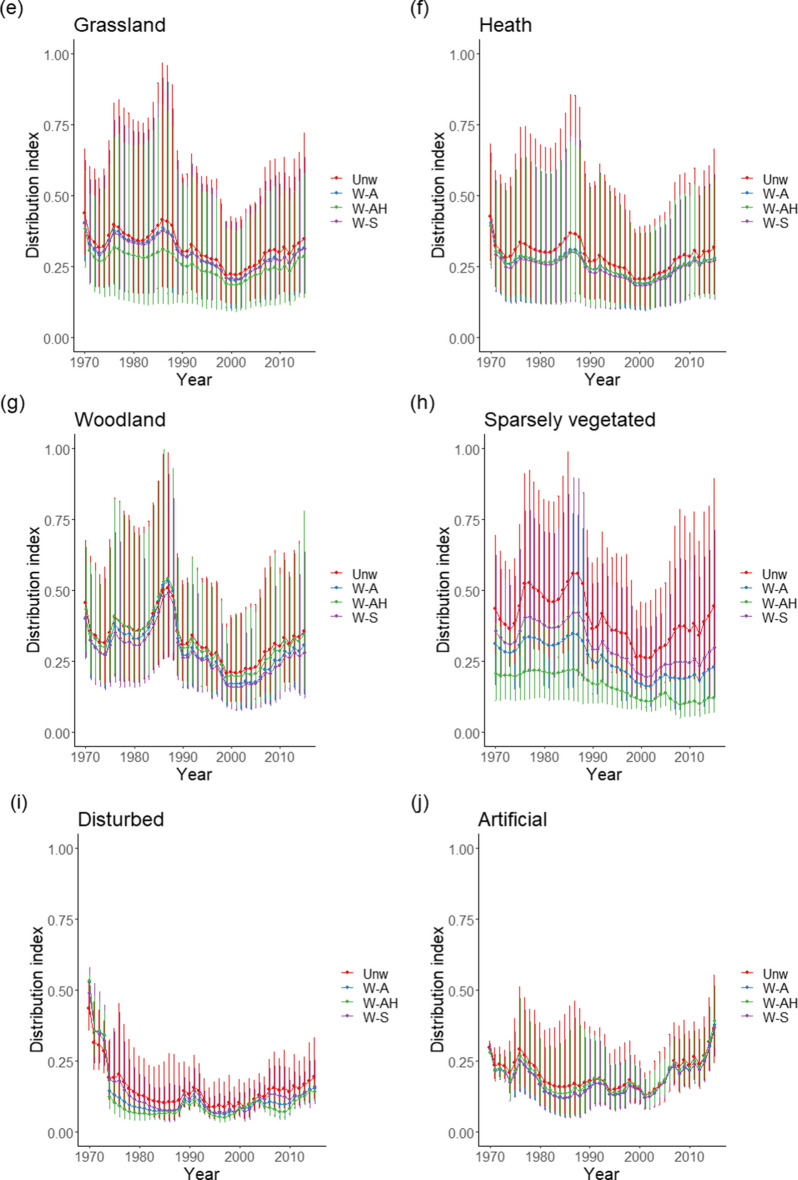
Trends in bryophyte Distribution Index by EUNIS level 1 habitat **a** Marine, **b** Coastal, **c** Freshwater, **d** Mires and bogs, **e** Grassland, **f** Heath, **g** Woodland, **h** Sparsely vegetated, **i** Disturbed, and **j** Artificial for the four weighting methods Unw—unweighted, W-S—weighting split evenly across habitats the species occurs in, W-A—weighting according to relative abundance of species per habitat, and W-AH—weighting according to relative abundance per habitat and the relative areas of habitat. Vertical lines represent the geometric standard deviations

This pattern was not consistent across all habitat types and the magnitude of the differences between methods was also not consistent (Fig. 2, Table 2), but all contrasts with the unweighted methods were significant (*p* < 0.001). The method of weighting using both abundance and area gave the lowest Distribution Index estimates for the habitat types Marine, Freshwater, Grassland, Sparsely vegetated, and Disturbed, as well as close to the lowest for Coastal. However, this method was the least different from the unweighted method for Woodland and Artificial. For Mires and bogs, the different weighting methods were indistinguishable in the effect, but all methods provided Distribution Index scores substantially less than the unweighted methods. For Artificial habitats weighting resulted in Distribution Index scores that were 5 to 11% lower than the unweighted method, but for Sparsely vegetated the weighting method using abundance and area provided Distribution Index estimates around 60% less than the unweighted methods. On average across the methods, habitat types Grasslands, Heath, Woodland, and Artificial had Distribution Index estimates 9 to 12% lower than the unweighted mean, habitat types Marine, Coastal, Freshwater, Mires and bogs and Disturbed had Distribution Index estimates 15–20% less and Sparsely vegetated habitats 40% less.

**Table 2:** Mean Distribution Index estimates from individual habitat mixed models comparing the four weighting methods: unweighted occupancy scores (Intercept), W-S—weighting split evenly across habitats the species occurs in, W-A—weighting according to relative abundance of species per habitat, and W-AH—weighting according to relative abundance per habitat and the relative areas of habitat for bryophytes and lichens. Habitat codes A—Marine, B—Coastal, C—Freshwater, D—Mires and bogs, E—Grassland, F—Heath, G—Woodland, H—Sparsely vegetated, I—Disturbed, and J—Artificial. All contrasts have *p* < 0.001 except *p = 0.039

	A	B	C	D	E	F	G	H	I	J
Bryophytes										
Intercept (Unw)	0.316	0.326	0.211	0.306	0.320	0.288	0.331	0.401	0.151	0.206
W-S	− 0.026	− 0.067	− 0.025	− 0.055	− 0.024	− 0.040	− 0.051	− 0.096	− 0.016	− 0.022
W-A	− 0.050	− 0.022	− 0.042	− 0.059	− 0.022	− 0.031	− 0.035	− 0.147	− 0.027	− 0.022
W-AH	− 0.070	− 0.064	− 0.050	− 0.053	− 0.059	− 0.032	− 0.008	− 0.237	− 0.038	− 0.011
Lichens										
Intercept (Unw)		0.182		0.383	0.209	0.240	0.286	0.100		
W-S		− 0.032		0.031	− 0.006	− 0.006	− 0.023	− 0.023		
W-A		− 0.011		− 0.037	0.036	− 0.005	− 0.094	− 0.036		
W-AH		− 0.055		− 0.051	− 0.018	− 0.003*	− 0.072	− 0.031		

### Habitat specific trends—lichens

Analysing the difference across all habitat types revealed the same pattern as for the bryophytes, with all methods of weighting having significantly and substantially lower mean occupancies than the unweighted methods (Table 1). Distribution Index estimates for the weighting method including both relative abundance and habitat area (W-AH) were substantially lower than for the other two weighting methods, whilst that including only relative abundance (W-A) was lower than that for evenly splitting weights across habitats (W-S).

**Fig. 3:**
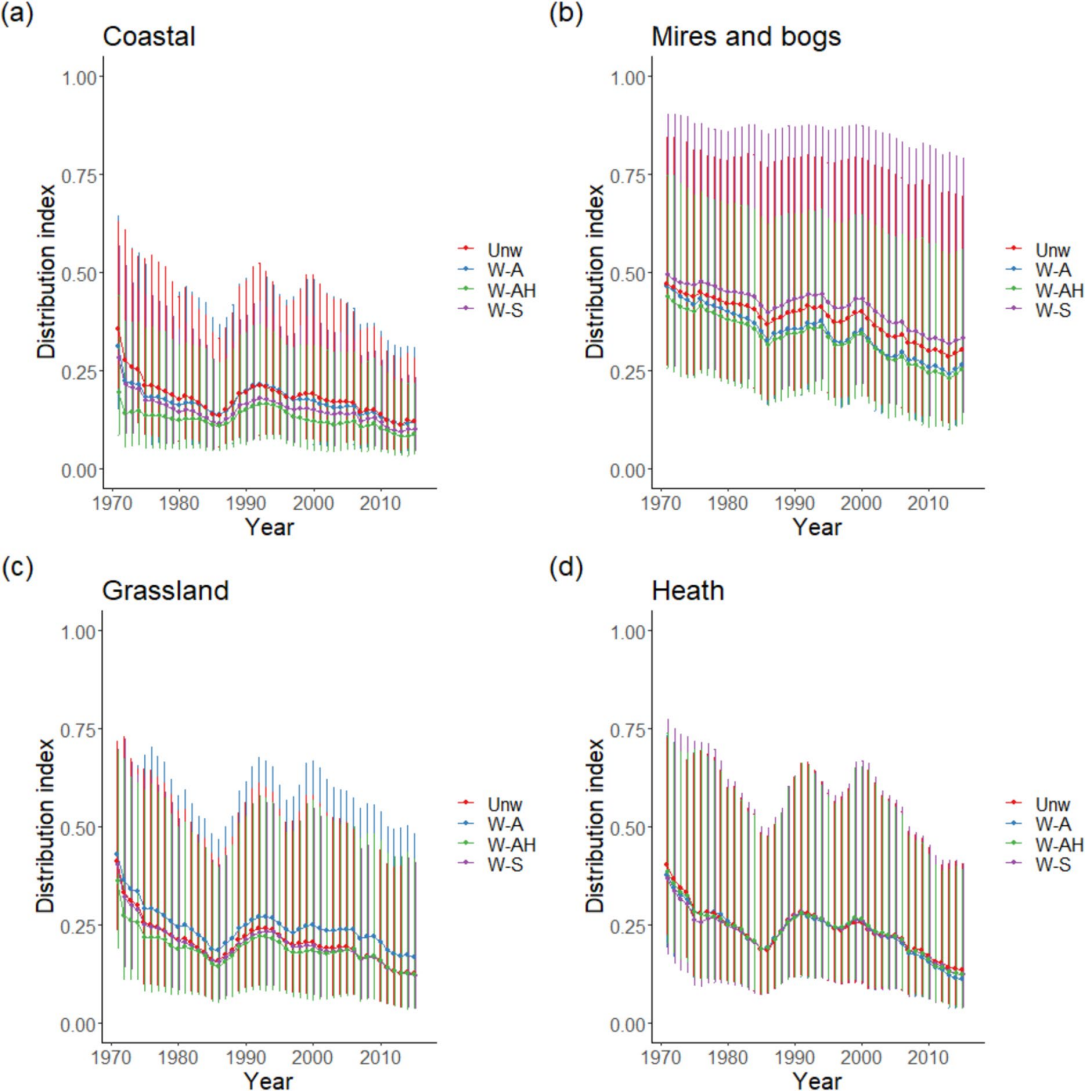

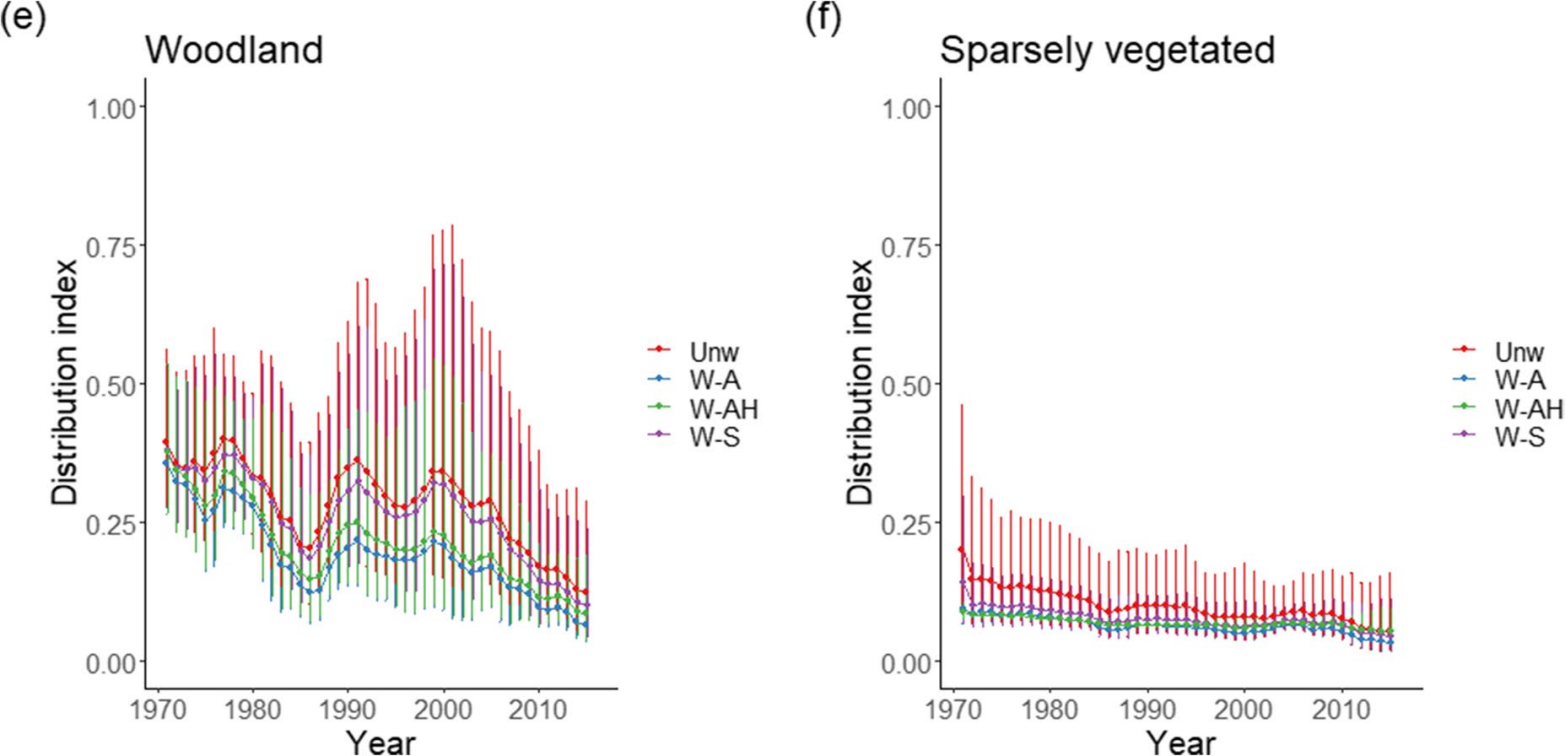
Trends in lichen Distribution Index by EUNIS level 1 habitat **a** Coastal, **b** Mires and bogs, **c** Grassland, **d** Heath, **e** Woodland, and **f** Sparsely vegetated for the four weighting methods Unw—unweighted, W-S—weighting split evenly across habitats the species occurs in, W-A—weighting according to relative abundance of species per habitat, and W-AH—weighting according to relative abundance per habitat and the relative areas of habitat. Vertical lines represent the geometric standard deviations

Again, the pattern was not consistent across all habitat types, with no habitat having the same ranking as the overall analysis (Fig. 3, Table 2). Weighting using abundance and area information (W-AH) produced the greatest reduction in mean Distribution Index for habitat types Coastal, Mires and bogs, and Grasslands, but weighting by abundance (W-A) only produced the greatest reduction for Woodland and Sparsely vegetated. However, for Mires and bogs, evenly splitting weights (W-S) increased the mean Distribution Index compared to the unweighted method, whilst using only abundance (W-A) did this for Grasslands. On average, across the methods, habitat types Mires and bogs, Grasslands, and Heath had a Distribution Index less than 5% lower than the unweighted mean, whilst habitat types Coastal, Woodland, and Sparsely vegetated had Distribution Index estimates more than 18% less.

### Long- and short-term trends

**Fig. 4:**
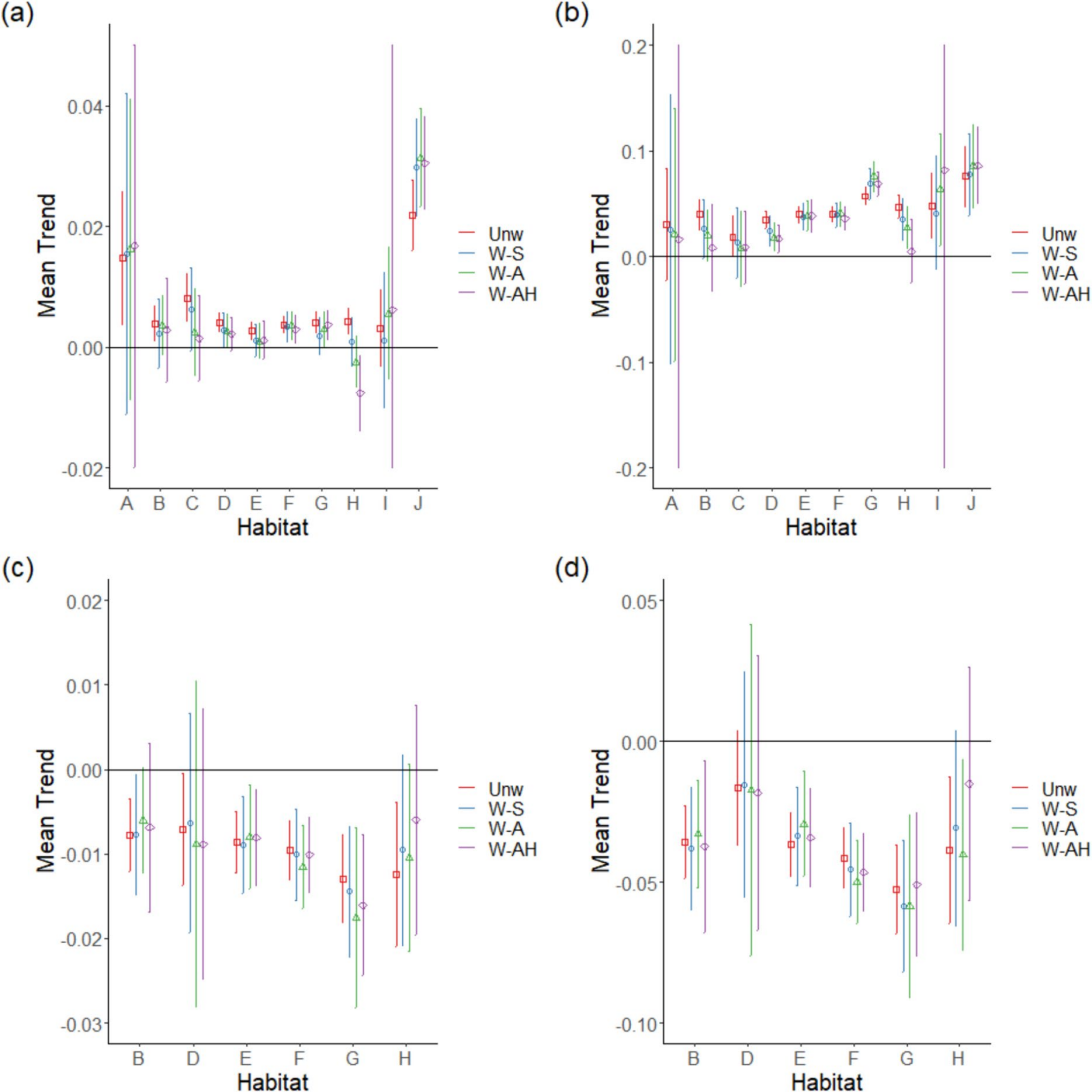
Mean **a** long-term and **b** short-term trends for bryophytes and mean **c** long-term and **d** short-term trends for lichens across EUNIS habitats according to the weighting method used: Unw—unweighted, W-S—weighting split across habitats, W-A—weighted according to relative abundance in different habitats, and W-AH—weighted according to relative abundance and relative area of habitats. Habitat codes are A—Marine, B—Coastal, C—Freshwater, D—Mires and bogs, E—Grassland, F—Heath, G—Woodland, H—Sparsely vegetated, I—Disturbed, and J—Artificial. Confidence intervals for weighting method W-AH for Marine and Disturbed bryophytes extend further than the axis limit. Vertical lines represent the 95% confidence intervals

Long-term trends for bryophyte species in Artificial habitats were more positive than for all other habitats except Marine (Fig. 4, Table 3). There was weak evidence for a consistent difference in means of methods (*p* = 0.059), with for most habitats a switch to weighting species across habitats resulting in more negative trends. This was especially true for Sparsely vegetated habitats, as the weighting became progressively more complicated there was a switch from an overall mean positive to a negative trend. This general pattern was not seen for bryophytes of Disturbed and Artificial habitats where more complex weighting made trends more positive. What was also noticeable was that as weighting was employed, positive trends for Marine, Coastal, Freshwater, Mires and bogs, and Grassland bryophytes became indistinguishable from zero as confidence intervals increased in size at the same time as the trends became less positive.

**Table 3:** Probability values from analysis of variance comparing habitats and weighting methods for long-term (LT) and short-term (ST) trends in bryophyte and lichen occupancy

	Habitat	Method	Interaction
Bryophyte long-term	< 0.001	0.059	0.584
Bryophyte short-term	< 0.001	0.413	0.632
Lichen long-term	0.054	0.992	0.999
Lichen short-term	0.001	0.991	0.999

For short-term trends in bryophytes, differences between habitat trends changed as the weighting method was changed (Fig. 4b). There was no difference between habitat trends for unweighted data, but splitting species evenly across the habitats they occurred in (W-S) identified Woodland bryophytes doing better than Freshwater, Mires and bogs, Grassland, and Heathland bryophytes. Including relative abundance (W-A) in habitats extended this list of habitats with bryophytes doing less well than Woodland bryophytes to include Coastal and Sparsely vegetated, whilst it also identified that bryophytes of Artificial habitats were doing better than Coastal, Freshwater, and Mires and bogs. Including habitat area (W-AH) did not add any further habitats to those doing less well than Woodland bryophytes but extended to list of habitats doing less well than those of Artificial habitats to include Grassland and Heathland bryophytes. There was no difference between mean trends between the weighting methods (Table 3), but for bryophytes of Coastal and Sparsely vegetated habitats, positive trends became indistinguishable from zero, as it did for two weighting methods for Disturbed habitats.

There was weak evidence that there were differences in long-term trends between habitats for lichens (*p* = 0.054, Table 3) with trends for Woodland lichens being more negative than those of other habitats (Fig. 4c). There was no difference in mean trends calculated with the different weighting methods, but including weighting meant that negative trends became zero for Coastal lichens for methods including abundance and area and for lichens of Sparsely vegetated habitats for all three weighting methods.

Analysis of short-term trends for lichens showed that Woodland lichens had significantly more negative trends than lichens of other habitats (Fig. 4d, Table 3). There were no differences between mean trends according to the weighting methods used, but negative trends for lichens of Sparsely vegetated habitats became indistinguishable from zero if the weighting involved even splitting lichen weights across habitats or it included both abundance and habitat area.

## Discussion

The analysis clearly shows that weighting species by some measure of their distribution across habitats has a clear impact on the conclusions that can be drawn from habitat trend data. This varied between habitats; for instance, the different weighting methods were indistinguishable for the bryophytes of Mires and bogs, but Sparsely vegetated saw a wide spread of overall trends data (Fig. 2). This may be a result of a relatively low number of generalists in Mires and bogs but a high proportion of generalists in Sparsely vegetated habitats, as evidenced by the reduction in weights between methods for the latter (Table 4). Two habitats saw very similar values for all methods, Woodland and Artificial. This may be a result of a greater degree of specialisation in these habitats and the impact of including habitat area actually increased the average weighting of species in these two habitats. The low areas of Marine, Coastal, Sparsely vegetated, and Disturbed in Scotland all resulted in low average weights in the analysis when habitat area was included in the weighting. Including generalist species in exactly the same way as specialist species in computing habitat trend data overestimates how well species are doing in that habitat as generalist species appear to be doing better, on average, than more specialised ones (e.g. Roth et al., 2021; Van Swaay et al., 2006; Wretenberg et al., 2006) or those of moderate generalism (Sullivan et al., 2016).

**Table 4:** Mean weights of the species contributing to habitat trends according to weighting method: Unw—unweighted occupancy scores, W-S—weighting split evenly across habitats the species occurs in, W-A—weighting according to relative abundance of species per habitat, and W-AH—weighting according to relative abundance per habitat and the relative areas of habitat for bryophytes and lichens. Habitat codes A—Marine, B—Coastal, C—Freshwater, D—Mires and bogs, E—Grassland, F—Heath, G—Woodland, H—Sparsely vegetated, I—Disturbed, and J—Artificial

	A	B	C	D	E	F	G	H	I	J
Bryophytes										
Species	2	28	15	83	96	111	73	48	6	7
Unw	1	1	1	1	1	1	1	1	1	1
W-S	0.171	0.255	0.342	0.354	0.348	0.373	0.354	0.305	0.321	0.529
W-A	0.197	0.348	0.306	0.373	0.281	0.392	0.379	0.279	0.336	0.529
W-AH	0.007	0.118	0.319	0.381	0.266	0.403	0.584	0.131	0.008	0.594
Lichens										
Species		12		5	16	18	8	3		
Unw		1		1	1	1	1	1		
W-S		0.357		0.257	0.414	0.414	0.458	0.567		
W-A		0.473		0.117	0.366	0.511	0.239	0.589		
W-AH		0.182		0.171	0.420	0.609	0.389	0.392		

Evenly splitting the contribution of a species to each of the habitats it occurs in is not ideal, but without further information it does at least solve the problems of generalist species having more weight across the analysis than specialist ones. Gradually including more information on abundance and habitat area makes logical sense as it means that it includes information on both frequency and on the area of contrasting habitats. For the analyses focusing on annual changes in occupancy scores, weighting has little impact on the size of the geometric standard deviations for bryophytes (< 3% difference between the methods) and reduces it slightly for the lichens (< 7%). In contrast, this is not the case for the analysis of overall trends, where the standard errors for the weighted methods are generally twice the size of those for the unweighted methods, and this rises to around six times higher for the W-AH method for the bryophytes. This latter effect is largely driven by habitats with few specialist species, particularly Marine and Disturbed, but the overall increase in standard errors does suggest that trends for specialist species appear to be more variable than those for generalists as the weighting downweights their control. It appears that mean trends for habitats calculated across species without weighting may give a false degree of confidence in their robustness. The results of this analysis suggest that species should not be included uncritically in more than one habitat when comparing habitat trends. The use of bird species across multiple habitats without weighting is therefore potentially an issue. For instance, Reed bunting and Yellow wagtail are included in both the Farmland and Wetland bird indicators, Skylark in both Farmland and Upland, Curlew, Redshank and Snipe in both Upland and Wetland bird indicators, and Lapwing in all three (Eaton & Noble, 2023; Noble & Barnes, 2023). Consideration ought to be given in how to deal with this and weighting is a logical way forward.

### Caveats

The analysis is somewhat crude as it amalgamates everything to EUNIS level 1 (10 habitats) but arguably reporting at a finer scale than this is unlikely, and the finer the cut, the fewer species are involved. Subtleties such as the occurrence of species in specific communities or subcommunities in the National Vegetation Classification are therefore lost, and there may be issues in the amalgamation of data across communities up to EUNIS level 1 that could be improved. However, the approach does clearly show that weighting the occupancy trends data appropriately is important.

The relatively low numbers of terricolous lichens left in the data set once the National Vegetation Classification information was merged with trend data meant that the analysis of patterns for only 25 species has little power to assess the response of this group of species to different methods. However, it does illustrate the challenges involved in trying to get a fine scale understanding of biodiversity trends and where the analysis becomes compromised by having too few species within a species group. It would not stop weighting in practice if a robust method was available for more species-rich groups that could be applied to more species-poor ones. It would be useful to repeat this analysis for other groups where it would be possible to replicate at least some of the different weighting methods.

Finally, the method developed here integrating abundance within habitats and habitat area (W-AH) does not account for changes in habitat area. Accounting for area and species trends in the same metric needs further work.

### Operationalising the approach

The specific approach outlined here can only work with bryophytes, lichens, and vascular plants. Even then, the National Vegetation Classification data is now comparatively old (1980s and earlier) and most of the sampling was associated with high-quality habitats, so the frequency of alien species is poorly represented for these two reasons. A substantial number of species may, therefore, not be amenable to this approach.

For all species, two alternative approaches are possible. The first would be to develop the statistical approach of Redhead et al. (2016), which identified associations between butterfly species and woodland habitats, by taking it further. Their approach could be extended to all habitats and then the relative associations used to generate weights to distribute individual species trends across habitats rather than allocating them in an unweighted fashion to all the habitats they were positively associated with (Burns et al., 2018; Hayhow et al., 2016). The data-hungry approach of Redhead et al. (2016) could be circumvented by focusing on species records with a precise location (1–10 m) that could be linked directly to a habitat map to give the number of records per habitat. Their approach could be used directly in place of the most complex weighting method used here. It does, however, limit the data used to create the weights to recent years where high-precision GPS data is available. It may also not be possible to work this out for species where location data is withheld due to their rarity. The issue of habitat weighting could be circumvented by focusing only on analysis with precise location data; however, that would limit the assessment of trends to only recent years.

Secondly, an expert-based approach could be used where other data are not available, which would include most taxa recorded as individual records. This would not be needed for sample-based approaches measuring community composition, which are used most frequently for bats, birds, butterflies, moths, and plants (Burns et al., 2018) and which can be analysed for habitat level changes directly. Experts on a group of species could be asked to estimate the relative chances of sampling a species in different habitats, accounting for differences of visibility in different circumstances. This could then be used with the habitat area data to create a final weighting for use in analysis. This second step avoids the difficulty of experts in estimating how many/much of a species is present across the different habitats.

### Biological significance for bryophytes and lichens

The trend analysis results are quite different from those reported in Pakeman et al. (2022) as only terricolous bryophytes and lichens are represented in the analysis here. The long-term trends for terricolous bryophyte species from Artificial habitats were always higher than the majority of habitats (Table 2), which was not the case in the analysis of all species (Pakeman et al., 2022). Including habitat area in the weighting also identified that bryophytes in Sparsely vegetated habitats were doing worse than those in Mires and bogs and Woodlands as well as in Heathlands as identified by Pakeman et al. (2022). The better short-term performance of bryophyte species in Woodlands was, however, a feature of both approaches.

The low number of lichen species in the analysis likely contributed to the finding of no differences between the trends for lichens from different habitats. However, the trends were all uniformly negative across habitats and significantly so for some habitats (Grassland, Heath and Woodland for short- and long-term trends, Coastal for long-term only, Fig. 4) indicating that terricolous lichens are performing poorly in these habitats. This may be associated with decreasing light levels, as vegetation has become denser or taller, as shown in the decline in Ellenberg light score for lichens across Scotland (Pakeman et al., 2022).

## Conclusions

The way species data is weighted in creating habitat level trend clearly has an impact in the calculation of trends within habitats, but those impacts differ somewhat between different habitats. Leaving the species unweighted is unsatisfactory as it means that generalist species are double counted, or more, and so their dynamics dominate the picture. This potentially smooths out the differences between habitats and, if generalists are doing well, create a too optimistic picture and vice versa. Reducing the weights of species in proportion to the number of habitats they occur in is simple but again is unsatisfactory as it weights a 99:1 distribution of a species between two habitats the same as a 50:50. However, this method has an overall useful effect of reducing the influence of generalists so that their total weight in the analysis is the same as more specialist species and most distributions of species between habitats will not be as extreme as 99:1.

Allowing the weights to be dependent on abundance or abundance plus habitat area gets closer to simulating the results of habitat monitoring. Including area gets round the potential issue of a species being very abundant in a rare habitat but being found across a more common habitat as well such that the trend in that species depends more on what is going on in that common habitat. However, none of these methods can cope with the situation where trends in different habitats are widely different.

Constructing metrics to assess how habitats are being affected by external drivers is important. Constructing them on the basis of individual species’ trends offers a potential route to do this without habitat focused monitoring. However, the analysis in this paper shows that there is a challenge in constructing the metrics if the breadth of species’ distributions varies between species, i.e. the species include both specialists and generalists. If trends differ between specialists and generalists, as they do, for example, for butterflies and woodland birds (Hayhow et al., 2019), then this may create bias in the indicators unless there is some attempt to weight species so that the influence of specialists and generalists is the same across all habitats. Without some correction for bias, it is possible that conservation policies and actions may not be optimal as the information on trends is not reflective of what might be occurring to the specialist species within a habitat. We would recommend at least some assessment of correcting for these biases in the production of future metrics and where possible incorporate relative abundance in different habitats and the relative area of habitats.

## Data Availability

No datasets were generated or analysed during the current study.
